# Surgical management of chronic Stevens-Johnson syndrome

**DOI:** 10.3389/fmed.2025.1669189

**Published:** 2025-11-07

**Authors:** Aastha Garg, Abha Gour, Nidhi Chauhan, Mehak Sapra, Virender Singh Sangwan

**Affiliations:** 1 Department of Cornea & Anterior Segment, Dr. Shroff’s Charity Eye Hospital, New Delhi, India; 2 Department of Cornea & Anterior Segment, Centre for Unknown and Rare Eye Diseases, Dr. Shroff’s Charity Eye Hospital, New Delhi, India; 3 Eicher Shroff Centre for Stem Cell Research, Dr. Shroff’s Charity Eye Hospital, New Delhi, India

**Keywords:** Stevens-Johnson syndrome, ocular surface stabilisation, punctal occlusion, mucous membrane grafting, amniotic membrane transplantation, COMET, keratoprosthesis, cataract surgery

## Abstract

Stevens-Johnson Syndrome (SJS) is a rare but severe mucocutaneous disorder that often leads to chronic ocular complications, requiring a comprehensive and multidisciplinary approach to management. This review outlines the pathophysiology and long-term ocular sequelae of SJS and discusses evidence-based strategies for stabilizing the ocular surface and restoring visual function. Key interventions include punctal occlusion to address tear film instability, mucous membrane grafting (MMG) for lid margin reconstruction, and amniotic membrane transplantation (AMT) to promote epithelial healing and reduce inflammation. Cultivated oral mucosal epithelial transplantation (COMET) and minor salivary gland transplantation (MSGT) serve as advanced options for restoring ocular surface function in severe cases. The correction of cicatricial entropion, often seen in chronic SJS, involves techniques such as anterior lamellar repositioning with or without grafting, which restore lid alignment and reduce ocular trauma. Visual rehabilitation through cataract surgery or keratoprosthesis demands careful preoperative planning due to the fragile ocular surface and heightened risk of postoperative complications. A tailored, staged management plan focused on surface preservation, structural correction, and visual restoration is essential to improve outcomes and quality of life for patients with chronic ocular SJS.

## 1 Introduction

Stevens-Johnson Syndrome is a rare but severe mucocutaneous disorder characterized by a hypersensitivity reaction commonly triggered by systemic medication or viral infections. The progression of the disease consists of both acute and chronic phases. In the acute stage, it presents with epidermal necrolysis and skin sloughing, creating a medical emergency that poses a significant risk to life. Prompt identification and suitable interventions, both locally and systemically, are crucial for survival and the prevention of corneal blindness. Ophthalmic symptoms should be swiftly recognized and addressed to prevent serious ocular complications during the chronic stage. Nevertheless, ophthalmologists predominantly see patients in this phase, as acute management is usually handled by dermatology or burn units, resulting in a lack of ophthalmic assessments in many instances. Addressing recurrent flare-ups during the chronic stage is particularly challenging and often proves to be ineffective.

While deemed uncommon in Western literature, the prevalence of this condition in India is significant, mainly due to the extensive use of drugs like sulphonamide antibiotics. The mortality rates associated with the disease differ, ranging from 1 to 5% in cases of SJS and 25 to 35% in instances of TEN (1). In children, the mortality rate may reach as high as 7.5% (1). Management in the acute stage involves topical and systemic immunosuppression along with amniotic membrane transplant. Patients who were adequately managed in the acute phase showed better outcomes in the chronic management stage.

**Figure 1:**
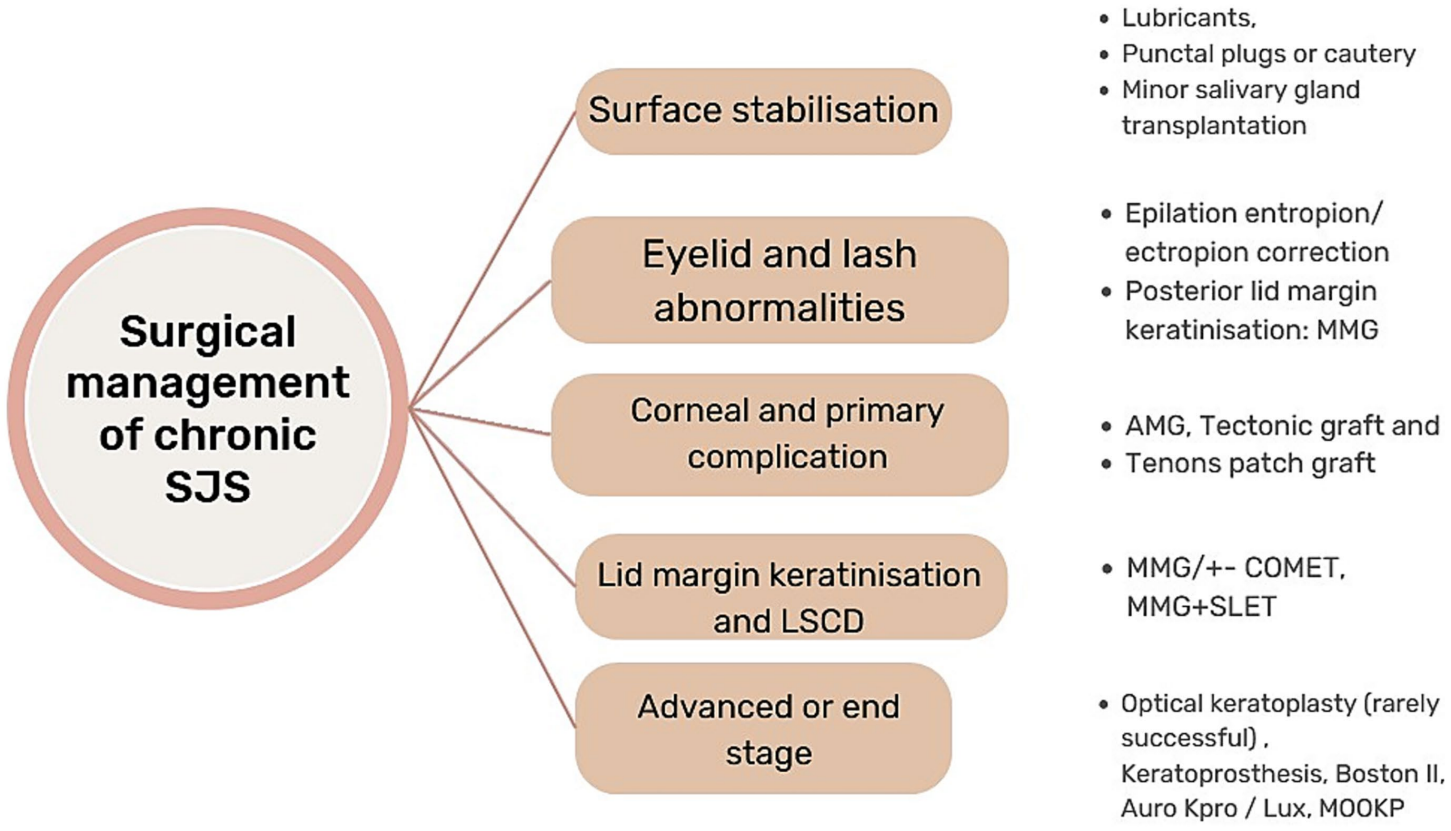
Flow chart illustrating a stepwise surgical approach to chronic SJS management.

The primary aim in managing chronic disease remains the stabilization of the ocular surface and patient comfort by preventing the formation of epithelial defects and surface keratinization, a step-by-step process that has been discussed in Figure 1. This article discusses the long-term management of SJS cases in the chronic stage, patient comfort, and visual rehabilitation.

## 2 Method of literature search

**Figure 2:**
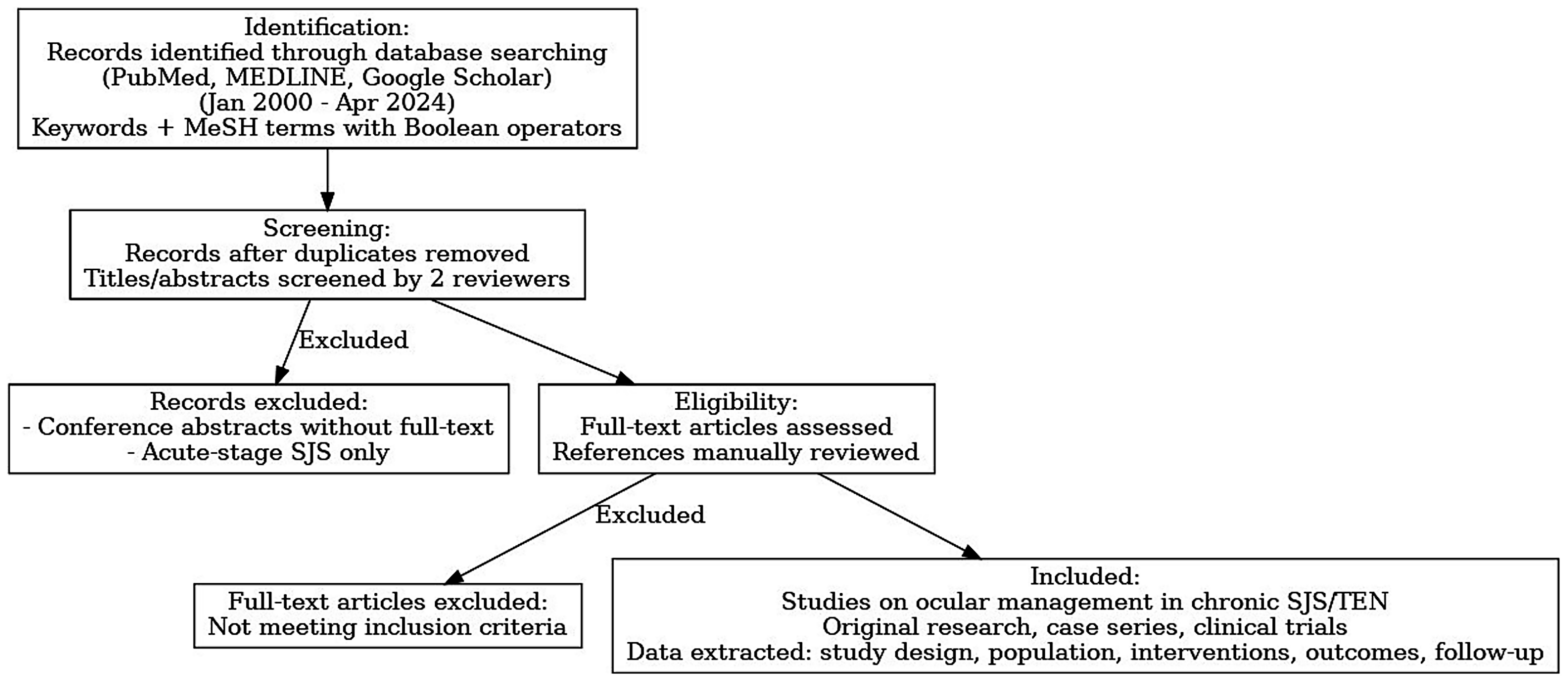
Flow diagram of our review of literature process.

A comprehensive review of literature (Figure 2) search was conducted across three major electronic databases: PubMed, MEDLINE, and Embase, for articles published from January 2000 to April 2024. The search strategy employed combinations of the following keywords: “Stevens-Johnson Syndrome,” “ocular surface disease,” “chronic ocular SJS,” “entropion correction,” “mucous membrane grafting,” “amniotic membrane transplantation,” “dry eye,” “keratoprosthesis,” “visual rehabilitation,” and “minor salivary gland transplantation.” Boolean operators (AND/OR) were used to refine search results.

Inclusion criteria were: (1) original research articles, case series, or clinical trials focusing on ocular management in chronic SJS or TEN; (2) articles published in English; and (2) all human, animal, and laboratory studies. Exclusion criteria included: (1) conference abstracts without full-text availability; and (2) articles that exclusively addressed acute-stage SJS without chronic ophthalmic follow-up.

Two independent reviewers screened titles and abstracts. Full-text articles were retrieved for eligible studies, and references of included articles were manually reviewed to identify any additional relevant studies. Disagreements were resolved by consensus. Data extracted included study design, patient population, intervention types, outcome measures, and follow-up duration.

A total of 57 studies were included in this review. Interventions evaluated across the literature comprised punctal occlusion (4 studies), mucous membrane grafting (MMG; 15 studies), amniotic membrane transplantation (AMT; 7 studies), cultivated oral mucosal epithelial transplantation (COMET; 9 studies), Simple limbal epithelial transplantation (SLET; 2 studies), minor salivary gland transplantation (MSGT; 12 studies), keratoprosthesis (KPro; 8 studies), and tarsoconjunctival pedicle graft (TPG; 3 studies). This distribution provides an overview of the evidence base for different surgical and medical approaches in the ocular management of chronic Stevens-Johnson syndrome and toxic epidermal necrolysis.

## 3 Stabilization of the ocular surface

Stevens-Johnson syndrome can result in significant ocular surface damage, including severe dry eye due to the loss of the tearing reflex, which ultimately leads to keratinization and corneal scarring. Managing dry eye in these patients typically involves controlling the surface inflammation and supplementing artificial tears with topical lubricants and autologous serum. The following section includes key techniques used in the stabilization of the ocular surface.

### 3.1 Punctal occlusion

Lacrimal punctal occlusion has been recognized as a simple, safe, and effective approach for treating aqueous tear deficiency and many ocular surface disorders. Punctal occlusion can cause the retention of the tear film on the ocular surface for a longer duration. Prolonged contact of tears allows essential tear components, such as vitamin A, epidermal growth factor (EGF), and transforming growth factor (TGF-alpha), to help maintain tear homeostasis. While vitamin A suppresses keratinization and promotes differentiation of corneal epithelial cells, EGF and TGF-alpha promote corneal epithelium turnover and wound healing (3).

The procedure involves injecting 2% lidocaine hydrochloride into the skin and conjunctiva surrounding the punctum to provide local anesthesia. The ophthalmic monopolar cautery tip is then inserted into the distal-most portion of the canaliculus through the punctum. Cauterization is then performed until the surrounding tissue shrinks, blanches, and turns white. It usually lasts 5–10 s. Following the application of this antibiotic eye ointment. Postoperatively, an antibiotic eye ointment is applied, and preservative-free medications are recommended to minimize ocular surface toxicity. Although generally well tolerated, potential complications include pain; occasional granuloma formation, which responds to topical steroid application; and lacrimal punctal recanalization.

Clinical studies have shown stabilization of the ocular surface as well as improvement in visual acuity post-punctal cautery. Iyer et al. demonstrated that punctal cautery alone in a moderate to severe dry eye improved or stabilized the visual acuity, along with improvement of the ocular surface status in >90% of the eyes (2). In another study by Tuberville et al., where punctal occlusion was performed in 32 eyes with tear deficiency syndromes, including patients with Stevens-Johnson syndrome, subjective symptomatic improvement was noted in 97% of the eyes (4). Kaido et al. conducted a retrospective study to assess the efficacy of lacrimal punctal occlusion in managing chronic ocular surface disease associated with Stevens–Johnson Syndrome (SJS). They found significant improvement in symptoms in 61.3% of eyes, with concurrent improvements in visual acuity, tear retention, and ocular surface health, as measured by Schirmer tests, tear clearance rates, and Rose Bengal and fluorescein staining scores. No patients experienced worsening symptoms, and complications were minimal. Therefore, punctal occlusion is a beneficial adjunct therapy for enhancing tear film stability and ocular surface integrity in chronic SJS (3).

### 3.2 Mucous membrane grafting (MMG)

**Figure 3:**
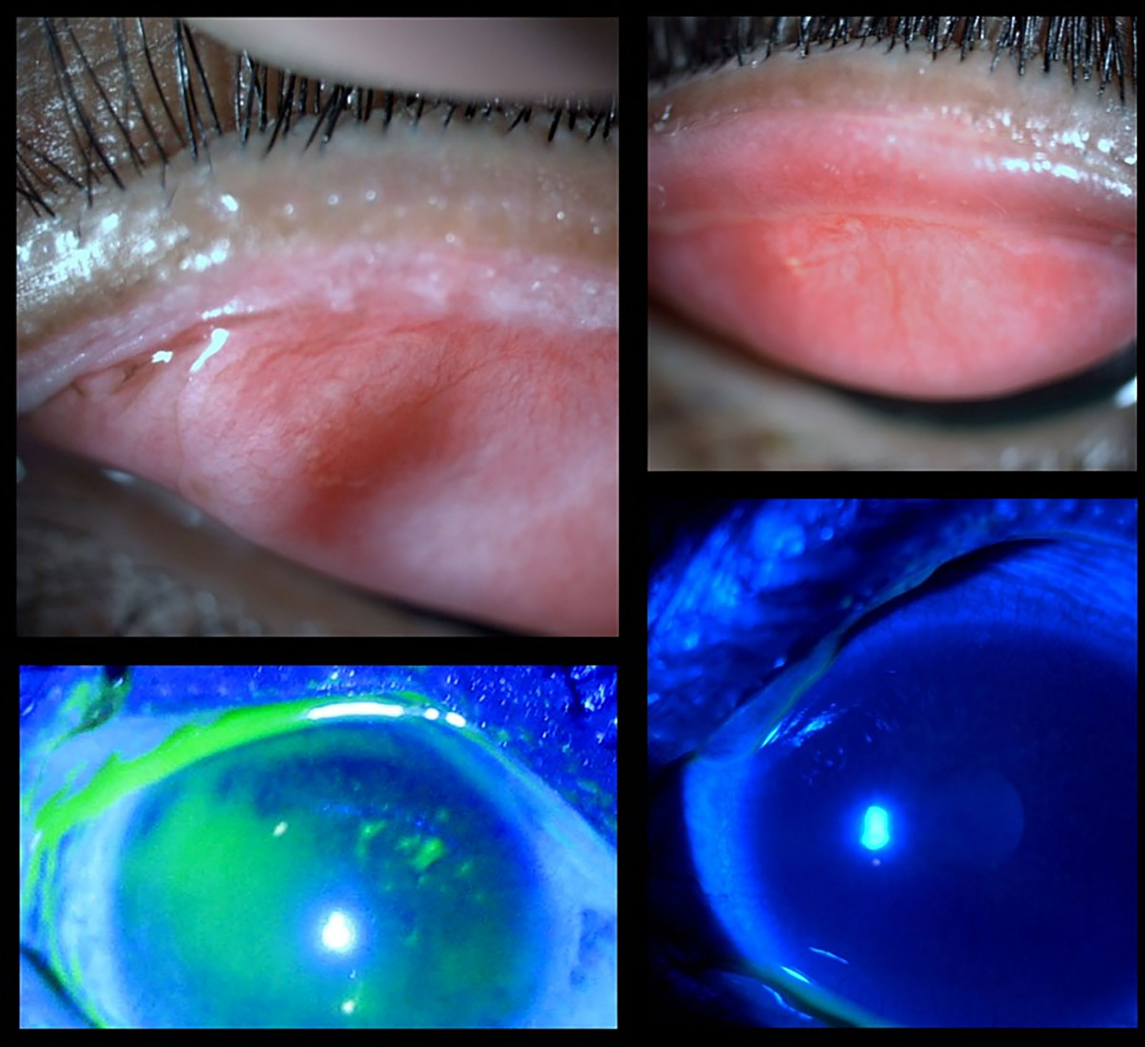
Clinical images of an SJS patient pre- and post-mucous membrane grafting. (Top left) Posterior migration of the mucocutaneous junction with lid margin keratinization. (Bottom left) Fluorescein-stained image showing punctate staining and an irregular surface due to the lid wiper effect. (Top right) Post-MMG image showing improved lid margin architecture. (Bottom right) Fluorescein-stained image indicating a more stable and healthier ocular surface following grafting.

Lid margin keratinization (LMK) is frequently observed and a vision-threatening sequelae in chronic SJS/TEN syndrome. It represents a protective response to chronic inflammatory stress. Various hypotheses have been proposed for LMK. Epidermalization of the adjacent skin, leading to the disruption of the mucocutaneous junction (MCJ) barrier during acute SJS, may allow skin epidermal cells to invade the conjunctival side; severe meibomian gland dysfunction (MGD) may also contribute through immune reactions; alterations in the microbiota on the lid margin can lead to further glandular disruptions; and the mechanism of squamous metaplasia involves the transformation of conjunctival epithelium into keratinized squamous cells due to the downregulation of anti-inflammatory proteins like EP3 and the upregulation of keratinization markers like TGase1 (4). The exact incidence of lid margin keratinization in patients with chronic ocular Stevens–Johnson syndrome/toxic epidermal necrolysis (SJS/TEN) remains uncertain. However, in a cohort monitored from the acute to the chronic phase, LMK developed in 22 to 100% of patients, exhibiting varying levels of severity, with a median onset time of 3 months (5). If left unaddressed, LMK can lead to progressive lid-related keratopathy and thus irreversible vision loss. By restoring a smooth lid margin, mucous membrane grafting (MMG) helps in protecting the ocular surface, including limbal epithelial stem cells. MMG can preserve corneal clarity and vision and mitigate corneal vascularization and scarring (Figure 3). It acts as a physical barrier against the posterior migration of the mucocutaneous junction, thereby preventing further keratinization, which in turn reduces ocular surface inflammation by reducing the proinflammatory cytokines and increasing the anti-inflammatory ones. This may occur, possibly due to goblet cells in the oral or lip mucosal graft (6). A study performed by Gurumurthy et al., evaluated the changes in ocular surface pre- and post-MMG. Researchers found that chronic SJS is characterized by a distinct pro-inflammatory, profibrotic, and anti-apoptotic cytokine environment, with significantly elevated levels of cytokines such as GM-CSF and IL-17A, and markedly reduced levels of antifibrotic markers like IP-10 and IFN-*γ*. MMG resulted in notable clinical improvements, including increased goblet cell density and a partial normalization of cytokine levels (7). The studies suggest that MMG not only addresses the mechanical impact of LMK but may also help restore a healthier ocular surface milieu, supporting its role in managing chronic ocular complications of SJS.

The surgery is usually performed under general anesthesia (GA) to ensure better patient comfort. The surgery begins by placing stay sutures on the lip and eyelids to evert tissue for exposure. The keratinized lid margin is incised from the center at the gray line or close to the lash line (18–20 mm horizontally), and a rectangular epithelial strip is dissected off the tarsus, ensuring complete removal to ensure better graft uptake. The desired length of labial mucosa is harvested from the lower or upper lip, avoiding the midline and areas of mucosal scarring post the disease. The excess fat and salivary tissue are removed from the graft until translucent. It is then divided, positioned on the lid, and sutured with 7–0 polyglactin using continuous interlocking sutures. Fibrin glue or minimal sutures are used to secure the posterior edge of the graft to avoid corneal irritation. A bandage contact lens is placed to protect the cornea from discomfort (6).

A study by Pushker et al. compared the outcomes of fibrin glue (FG) and three cardinal sutures to continuous polygalactin sutures (PG) for MMG, then the patients in each group. While the recurrence of lid margin was 7.5% in both groups, recurrence of metaplastic lashes and graft displacement occurred in fewer eyes in the fibrin glue group compared to the polygalactin suture group. Operative time was also shorter in the FG group (39.5 min vs. 56 min). FG provides similar outcomes with shorter surgery time, making it a good alternative when cost permits (8).

Several studies have evaluated the efficacy of mucous membrane grafting (MMG) in managing lid margin keratinization (LMK) and associated ocular surface complications in Stevens–Johnson syndrome (SJS) patients. Iyer et al. (2) reported on 238 eyes (460 lids) of 138 patients with LMK (Grade ≥2), demonstrating that over 80% of eyes showed improved or stabilized BCVA and corneal staining, with more than 60% achieving improved Schirmer’s wetting. Revision MMG was required in 11.3% of cases due to graft shrinkage or recurrent keratinization. In a larger cohort, Iyer et al. (9) studied 393 eyes of 230 SJS/TEN patients and observed improvements in VA in 129 eyes, corneal staining in 130 eyes, and Schirmer I scores in 111 eyes, with an 8.4% recurrence of keratinization necessitating revision MMG. Earlier work by Iyer et al. (10) in 54 eyes, demonstrated that 92.6% of patients showed improvement or stabilization in comfort, hyperemia, corneal staining, and BCVA over a mean follow-up of 6 months. Long-term outcomes reported by Shanbhag et al. (11) in 100 eyes (81 adults, 19 pediatric) over a median 60-month follow-up revealed visual acuity gains, with pediatric eyes improving from 20/100 to 20/30 and adult eyes from 20/100 to 20/60. More recent data by Sinha et al. (12) in 59 patients (90 eyelids undergoing electroepilation and 41 eyelids undergoing eyelid resection) over 20–79 months showed success rates of 67.8% for electroepilation (requiring multiple interventions) and 100% for eyelid resection (with fewer interventions), though 10 eyelids required MMG following recurrence. Overall, MMG plays a critical role in mitigating the complications of LMK, improving ocular surface stability, and preserving vision in patients with chronic ocular SJS/TEN. The outcomes of MMG across multiple studies, highlighting improvements in visual acuity, corneal staining, Schirmer’s test scores, and recurrence rates, are summarized in Table 1.

**Table 1:** Summary of outcomes and complications from studies on mucous membrane grafting (MMG) in SJS patients.

Study	Reference	Sample size/Condition	Follow-up duration	Key outcomes	Complications/Recurrence
Iyer (2014)	(2)	238 eyes (460 lids) of 138 patients with LMK (Grade ≥ 2)	Minimum 3 months	>80% showed improved/stable BCVA and corneal staining; >60% had improved Schirmer’s wetting	Revision MMG in 11.3% due to graft shrinkage or keratinization
Iyer (2016)	(9)	393 eyes of 230 SJS/TEN patients	≥3 months	VA improved in 129 eyes; Corneal staining improved in 130 eyes; Schirmer I improved in 111 eyes	8.4% recurrence of keratinization; Revision MMG performed
Iyer (2010)	(10)	54 eyes	6 months (mean)	92.6% showed improvement/stabilization in comfort, hyperemia, staining, and BCVA	Not reported
Shanbag (2020)	(11)	100 eyes (81 adults, 19 pediatric)	60 months (median)	VA improved: Pediatric (20/100 to 20/30), Adults (20/100 to 20/60)	Not reported
Sinha (2024)	(12)	59 patients (90 eyelids - electroepilation; 41 eyelids – eyelid resection)	20–79 months (mean)	Electroepilation success 67.8% (with multiple interventions); Eyelash resection success 100% (fewer interventions)	More procedures needed with RF; 10 eyelids required MMG after recurrence

VA: Visual Acuity; BCVA: Best Corrected Visual Acuity; LMK: Lid Margin Keratinization; MMG: Mucous membrane grafting.

### 3.3 Ocular surface reconstruction

- Amniotic membrane transplantation (AMT)

It is a well-established therapeutic option in the management of severe ocular surface disorders including chronic Stevens-Johnson Syndrome. Initially demonstrated in experimental animal models for reconstructing the corneal surface in cases of total limbal stem cell deficiency (LSCD), AMT has shown clinical success in treating partial limbal stem cell deficiency by promoting epithelialization and reducing inflammation and vascularization. In total LSCD, AMT is combined with allograft limbal transplantation to achieve better outcomes. AMT serves as a preparatory step that reconstructs the perilimbal stroma, thereby enhancing the success of allograft limbal transplantation in severe ocular surface diseases like Stevens-Johnson syndrome.

**Figure 4:**
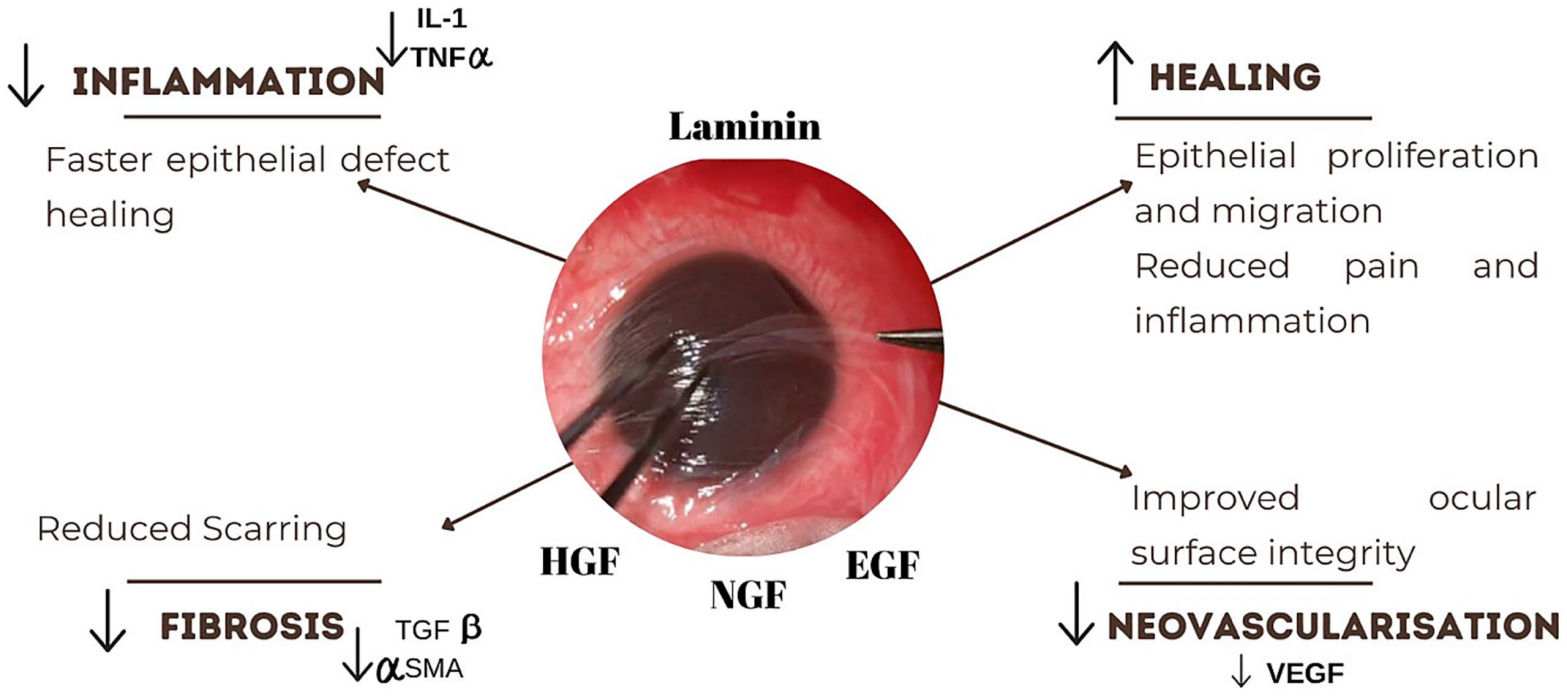
Mechanism of action of amniotic membrane graft. The graft reduces inflammation by downregulating IL-1 and TNF-*α*, promoting faster epithelial healing. It also stimulates epithelial proliferation and migration, alleviating pain and inflammation. Suppression of TGF-*β* and α-SMA levels helps reduce fibrosis and scarring.

The efficacy of AMT stems from its basement membrane, which mimics the conjunctival basement membrane, providing a scaffold that supports epithelial progenitor cells, limbal stem cells, and transient amplifying cells, thereby promoting epithelial proliferation and differentiation into goblet and non-goblet cells. It suppresses the inflammation by excluding the inflammatory cells and their protease activities. It inhibits transforming growth factor-beta (TGF-beta) signaling and myofibroblast differentiation, thereby reducing scarring and vascularization (Figure 4). These combined effects facilitate rapid re-epithelialization, maintain normal epithelial phenotypes, promote tissue remodeling, and reduce fibrosis (13).

The surgical technique usually involves removing the entire pannus on the ocular surface, including releasing symblepharon adhesions. The healthy episclera is exposed. The remaining bulbar and palpebral conjunctiva is used to form the fornix, using a double-armed silk suture that is passed through the conjunctivae to the periosteum of the orbital rim, exiting at the eyelid skin and tied. The entire denuded area is then covered with epithelium-basement membrane up which is then secured to the conjunctival edge with interrupted 8–0 Vicryl sutures that are placed just outside the limbus. The symblepharon rig is then inserted. Postoperatively, the treatment regime includes topical steroids tapered slowly over several weeks and topical lubricants (14).

Clinically, AMT has been shown to restore conjunctival integrity, deepen the fornices, prevent recurrence of symblepharon, and facilitate corneal epithelial regeneration, thereby improving ocular surface stability and reducing discomfort. Outcomes of AMT in the stabilization of the ocular surface are discussed in Table 2. Honavar et al. (14) used AMT with fornix sutures in 10 eyes, all from SJS patients, and found that fornix depth was achieved in 9 out of 10 eyes after a mean follow-up of 13.5 months; all eyes experienced full re-epithelialization except for one that required keratoplasty. Kheirkhah et al. (15) performed oral mucosal transplantation (OMT) combined with AMT and mitomycin-C (MMC) in 5 eyes, reporting their outcomes as part of an overall 84.4% complete success rate after a mean follow-up of 16.4 months, with better success observed in Grade III compared to Grade IV SJS cases. Solomon et al. (16) examined AMT alone in 2 eyes of a single patient, grouping their data into a broader cohort with a reported 70.6% complete fornix success rate over a mean follow-up of 37 months, noting that autoimmune cases had worse results. Tseng et al. (13) evaluated MMC combined with AMT in 4 eyes, finding only one eye developed partial motility restriction, while most others showed restored fornix depth and tear meniscus after a mean follow-up of 14.2 months, with MMC used to suppress inflammation.

**Table 2:** Reported outcomes of amniotic membrane transplantation (AMT) in SJS patients across various studies.

Study	Reference	Surgical method	Sample size (SJS eyes)	Success rate/Outcome (SJS subgroup)	Follow-up duration	Notes
Honavar et al. (2000)	(14)	AMT + fornix sutures	10 eyes (all SJS)	Fornix depth achieved in 9/10 eyes	Mean 13.5 months	All eyes had full re-epithelialization; 1 central melt required keratoplasty
Kheirkhah et al. (2013)	(15)	OMT + AMT + MMC	5 eyes	Not isolated, but part of the overall 84.4% complete success	Mean 16.4 months	Grade IV SJS cases included; better success in Grade III
Solomon et al. (2003)	(16)	AMT	2 eyes (1 patient)	Not specifically stated; grouped into 12/17 success rate	Mean 37 months	AMT alone achieved 70.6% complete fornix success overall; autoimmune cases had worse results
Tseng et al. (2005)	(62)	MMC + AMT	4 eyes	1 eye had a recurrence of partial motility restriction; others were successful	Mean 14.2 months	Used MMC to suppress inflammation. Good fornix depth and tear meniscus restored in most

AMT: amniotic membrane transplantation; OMT: oral mucosal transplantation; MMC: mitomycin-C.

Amniotic membrane transplantation also plays a crucial protective role in chronic SJS patients undergoing cataract surgery. These patients are at high risk of postoperative epithelial breakdown due to baseline surface instability. The surgery often triggers an inflammatory cascade, which results in persistent epithelial defects or sterile ulcers. Intraoperative application of AMT significantly reduces the risk of epithelial complications, improves corneal clarity, and supports visual recovery. Further methodologically robust studies are required to thoroughly evaluate the efficacy of amniotic membrane transplantation (AMT) in promoting ocular surface stabilization and improving visual outcomes following cataract surgery. While there are no specific clinical published data solely on the use of AMT in cataract surgery, the authors have consistent clinical experience with the use of intraoperative AMT as a protective adjunct during cataract surgery in chronic SJS to mitigate postoperative epithelial breakdown and facilitate visual rehabilitation.

- Cultivated oral mucosal epithelial transplantation (COMET)

**Figure 5:**
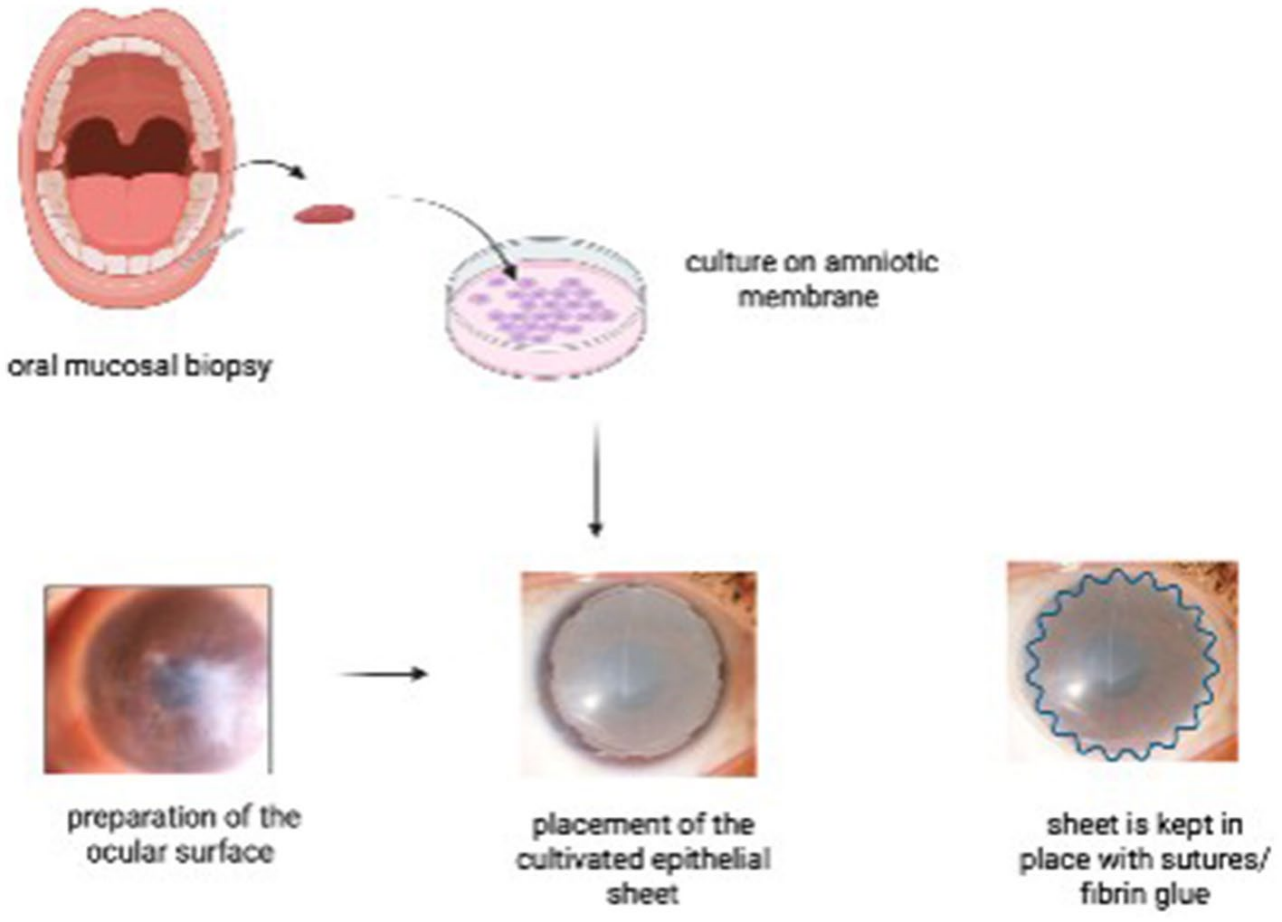
Steps of cultivated oral mucosal epithelial transplantation (COMET). An oral mucosal biopsy is harvested from the patient’s buccal mucosa and cultured on an amniotic membrane. In a second surgical stage, the ocular surface is prepared, and the cultivated epithelial sheet is transplanted onto the recipient bed, secured with sutures and/or fibrin glue.

It involves harvesting a small biopsy from the patient’s buccal region, which is then cultivated on an amniotic membrane substrate for 7–14 days to form a multilayered epithelial sheet. Epithelial differentiation is achieved either by co-culture with mitomycin C-inactivated 3 T3 fibroblasts. The air-lifting technique is used to promote stratification (17). Once cultivated, the sheet is transplanted onto the ocular surface following surgical lysis of symblepharon and fornix reconstruction. The COMET graft acts as a non-keratinized autologous epithelial substitute that restores the fornix architecture, thereby preventing recurrence of adhesions and supporting ocular surface stability (Figure 5) (18). A novel feeder-free and serum-free (FFSF) culture system for oral mucosal epithelial transplantation was demonstrated by Nakamura et al., which emphasized a zoonotic infection-free and clinically safe medium for culture. The study confirmed the generation of functional epithelial sheets containing p75(+) holoclone-type progenitor cells (19).

It re-epithelializes the damaged conjunctiva, which acts as a barrier against conjunctival ingrowth and prevents further scarring and fibrosis. It promotes healing without eliciting an immune response. The outcomes of COMET in SJS patients are discussed in Table 3. Nakamura et al. (20) reported on 11 eyes with 95% experiencing some visual improvement and 53% maintaining gains at 36 months, alongside a stable ocular surface with reduced neovascularization and symblepharon over a mean 55-month follow-up. Ang et al. (21) included 7 eyes with 90% achieving more than two lines of visual gain and a stable ocular surface at a 12.6-month mean follow-up. Sotozono et al. (22) assessed 3 eyes showing significant BCVA improvement with complete epithelialization and a median follow-up of 23.3 months. Aziza et al. (23) reported 16 eyes with LogMAR BCVA improving from 1.9 to 1.3, achieving a stable surface and deep fornix for contact lens placement over a mean 6.4 years of lens wear. Gopakumar et al. (24) described 11 eyes with 67% full and 33% partial epithelization, noting a stable surface with complete epithelialization at an 18-month mean follow-up. Sotozono et al. (25) covered 21 eyes with significant visual improvement over 24 weeks, alongside a stable surface and improved ocular surface grading score at a 28.7-month median follow-up. Inatomi et al. (26) evaluated 12 eyes with 67% remaining stable and transparent with more than two lines of visual gain, and a stable surface in 67% at 20 months on average. Inatomi et al. (27) (PKP + COMET) reported improvement to 20/125 with stable ocular surface after combined PKP and COMET over 32.5 months of follow-up. Nakamura (28) included 20 eyes, all showing improvement and a stable ocular surface in all cases at a 13.8-month mean follow-up, with abbreviations noting COMET as cultivated oral mucosal epithelial transplant, CL as contact lens, and PKP as penetrating keratoplasty. COMET is valuable in SJS patients where conventional grafts may fail due to severe inflammation and immune-mediated damage (28).

- Simple limbal epithelial transplantation

**Table 3:** Outcomes of cultivated oral mucosal epithelial transplantation (COMET) in SJS patients.

Study	Reference	SJS eyes	Visual improvement (%)	Ocular surface stabilization	Follow-up
Nakamura (2011)	(20)	11	95% (any), 53% (at 36 months)	Stable ocular surface, reduced neovascularization, and symblepharon	55 months (mean)
Ang (2006)	(21)	7	90% (>2 lines)	Stable ocular surface	12.6 months (mean)
Sotozono (2014)	(22)	3	Significant BCVA improvement	Stable ocular surface and complete epithelization	23.3 months (median)
Aziza (2024)	(23)	16	LogMAR BCVA improved from 1.9 to 1.3	Stable ocular surface, deep fornix for CL placement	6.4 years (mean CL-wear)
Gopakumar (2019)	(24)	11	67% full, 33% partial epithelialization	Stable ocular surface and complete epithelization	18 months (mean)
Sotozono (2013)	(25)	21	Significant improvement over 24 weeks	Stable ocular surface and ocular surface grading score	28.7 months (median)
Inatomi (2006), AJO	(26)	12	67% stable & transparent, >2 lines gain	Stable ocular surface in 67% of cases	20 months (mean)
Inatomi (2006), PKP + COMET	(27)	1	To 20/125	Stable ocular surface after PKP + COMET	32.5 months
Nakamura (2004)	(28)	3	All improved	Stable ocular surface in all cases	13.8 months (mean)

COMET: Cultivated oral mucosal epithelial transplant; CL: contact lens; PKP: Penetrating keratoplasty.

Although simple limbal epithelial transplantation (SLET) has emerged as a successful and widely adopted technique for unilateral limbal stem cell deficiency (LSCD), particularly in cases secondary to chemical injury, its outcomes in Stevens–Johnson syndrome (SJS) remain less favorable. In the study by Prabhasawat et al. (29), SJS was the second most common cause of LSCD (21.5%) and the leading cause of bilateral LSCD (35.3%), with six patients undergoing living-related alloSLET. Among these, only three achieved successful outcomes, while three failed, with SJS itself identified as a significant risk factor for graft failure (*p* = 0.035). Similarly, Shanbhag et al. (30) reported on 16 patients with SJS who underwent either live-related allo-SLET (*n* = 9) or cadaveric allo-SLET (*n* = 7), where recurrence of LSCD occurred in five eyes, four of which were attributed to SJS. The relatively high rates of failure in SJS are thought to be related to the hostile ocular surface environment, including chronic inflammation, severe dryness, adnexal pathology, and conjunctival cicatrization, which compromise the survival and function of transplanted limbal stem cells. These findings suggest that while SLET is promising for certain etiologies, its role in chronic SJS remains limited and requires cautious patient selection and adjunctive management strategies.

### 3.4 Minor salivary gland transplantation

Minor salivary gland transplantation involves harvesting a full-thickness graft containing labial or buccal mucosa with attached minor salivary glands, typically from the upper and lower lip (31). Donor site selection can be made accurate by guided minor salivary gland flow rate (MSGFR), with preference given to areas showing higher secretion ≥1.785 μL/min/cm^2^. A graft of 15-20 mm length is carefully dissected to include a sliver of orbicularis oris muscle for structural support (32). A recipient bed is prepared on the superior bulbar conjunctiva by making a horizontal incision of about 10 mm posterior to the limbus. The superior rectus muscle is isolated, and the graft is anchored to it by using absorbable sutures to ensure perfusion and stability (33). The mucosal surface is oriented outward, while the salivary glands face the sclera. Postoperative care includes topical steroids, antibiotics, and regular lubricant use.

The therapeutic mechanism of MSGT relies on the secretory function of the transplanted glands, which produce mucin-rich saliva continuously and endogenously. This fluid closely mimics natural tears in composition, containing electrolytes, immunoglobulins, lactoferrin, lysozyme, and mucins (MUC1, MUC16) (34). These components enhance tear film stability, reduce ocular surface inflammation, and promote epithelial healing. As a result, MSGT helps re-establish ocular surface homeostasis in SJS patients with absolute aqueous tear deficiency and severe keratinization, offering sustained relief in cases unresponsive to conventional therapies (33).

Table 4 summarizes outcomes from studies evaluating minor salivary gland transplantation (MSGT) in Stevens–Johnson syndrome (SJS)–related severe dry eye, highlighting consistent improvements in tear production and ocular surface health across cohorts and case reports. In a 2024 interventional series of 20 eyes with chronic SJS, Sharma et al. (35) reported a significant increase in Schirmer I test values at 1 year and broad ocular surface gains, including reductions in hyperemia, keratinization, epithelial defects, neovascularization, and corneal opacification, alongside improved palisades of Vogt; subjective symptom data were not detailed in the abstract, but the objective metrics showed clear benefit (all *p-*values ≤ 0.05, with Schirmer I *p* = 0.0004). Earlier clinical experience mirrors these findings: Wakamatsu and colleagues (34) described MSGT with labial mucosa for severe cicatricial dry eye (including SJS), noting better corneal clarity and neovascularization, increased Schirmer I scores, and improved best-corrected visual acuity; patients also reported relief of pain, photophobia, and foreign body sensation, indicating meaningful symptomatic improvement after surgery. Collectively, the evidence supports MSGT as a viable option for refractory, aqueous-deficient dry eye in SJS, with objective gains in tear metrics and ocular surface integrity and, in studies reporting patient-reported outcomes, notable symptom relief as well (31–33, 36).

**Table 4:** Outcomes of minor salivary gland transplantation (MSGT) in SJS patients.

Study	Reference	Sample	Schirmer I	Other ocular improvements	Subjective Relief
Sharma et al. (2024)	(35)	20 eyes with chronic SJS	Significant = 0.0004	↓ Hyperemia, keratinization, epithelial defect, neovascularization, opacification (all *p* < 0.05); ↑ Vogt’s palisades (*p* = 0.007)	Not reported
Sant’Anna et al. (2012)	(31)	19 patients with SJS	Improved in 73.7% (*p* = 0.0094)	↓ Neovascularization (*p* = 0.0005), ↑ Corneal transparency (*p* = 0.001)	↑ Pain (54.8%), photophobia (50.2%), foreign body sensation (53.6%) (*p* = 0.0167)
Vazirani et al. (2021)	(33)	Subgroup with SJS (not separated numerically)	Significant ↑ (*p* < 0.0036)	↓ Corneal staining, neovascularization, opacification	Visual acuity ↑ from 20/500 to 20/80 (3-year median)
Su et al. (2022)	(32)	SJS patients in 18 eyes (flow-based donor selection)	↑ from 0 mm to 4 mm (*p* < 0.05)	↓ Fluorescein staining; ↑ TBUT (quantified scores not reported)	58.8% reported symptom relief; greater relief when pre-op MSGFR >1.785 μL/min/cm^2^
Arboleda et al. (2023)	(36)	Single SJS case (case report)	↑ from 0 mm to 3 mm	↓ Keratinization; improved ocular surface wetness	Subjective comfort improved; successful Boston KPro Type 1 retention
Wakamatsu et al. (2017)	(34)	Subset with SJS (not separated numerically)	↑ from <1 mm (exact value not given)	↓ Neovascularization, ↑ Corneal clarity, and BCVA	Improved symptoms: pain, photophobia, foreign body sensation

TBUT: Tear breakup time; Kpro: Keratoprosthesis; BCVA: Best Corrected Visual Acuity.

### 3.5 Adnexal surgeries: entropion correction

The surgical correction of cicatricial entropion in Stevens-Johnson Syndrome (SJS) typically involves anterior lamellar recession (ALR) combined with mucous membrane grafting (MMG), tailored to address the inward rotation of the eyelid and associated lid margin keratinization (37, 38). Under local or general anesthesia, the anterior lamella—including skin and orbicularis muscle—is dissected and recessed away from the tarsus to correct the entropion (37). Keratinized conjunctival tissue at the lid margin is excised, and a mucous membrane graft, usually harvested from the labial or buccal mucosa, is sutured to the posterior lid margin to restore a non-keratinized epithelial surface (38–40). This not only repositions the lashes but also promotes ocular surface stability. In more severe cases, particularly involving the upper eyelid, a rigid graft of nasal septal cartilage with adherent mucosa may be used to structurally reinforce the lid and prevent re-inversion, as described by Callahan (41). Adjunctive procedures, such as eyelash resection or radiofrequency ablation are used in cases with persistent trichiasis (37, 40). Postoperative care includes topical antibiotics, corticosteroids, and intensive lubrication.

Table 5 summarizes the ocular surface improvement and visual acuity improvement in SJS patients observed post-adnexal surgeries (37, 38, 40). The surgical correction of entropion in patients with Stevens-Johnson Syndrome (SJS) remains a complex but essential component of ocular surface management. Given the progressive, cicatrizing nature of the disease, surgical strategies should prioritize tissue preservation, anatomical restoration, and long-term ocular surface protection. Techniques such as anterior lamellar repositioning, often combined with grafting methods like mucous membrane grafts when indicated, offer effective correction while minimizing additional trauma (38). A tailored, single-stage approach that addresses both functional and structural abnormalities can significantly reduce the burden of repeat interventions and improve patient outcomes. Careful surgical planning and an individualized approach are critical to achieving stability and symptomatic relief in this high-risk patient population.

**Table 5:** Ocular surface and visual acuity outcomes following adnexal surgeries in SJS patients.

Study	Reference	Sample (SJS eyes)	Entropion correction rate	Trichiasis resolution	Ocular surface improvement	VA improvement	Follow-up
Anklesaria et al. (2025)	(37)	11 eyes	100% at final follow-up	90% (1 repeat ALR, 1 lash resection)	87.5% symptom improvement	40%	14.8 months
Shree et al. (2023)	(38)	20 eyes	75% (no recurrence)	50% of eyes had trichiasis pre-op	70% had improved corneal staining	50%	10 months
Singh et al. (2021)	(40)	3 eyes	100%	83% (1 case managed with RFA)	All patients had reduced symptoms	Improved in all but one	12–18 months

ALR: Anterior Lamellar Resection; RFA: Radiofrequency Ablation.

## 4 Tectonic support

### 4.1 Management of perforations: tissue adhesive (TABCL), Tenon patch graft (TPG), patch grafts

Tenon patch graft (TPG) involves using autologous Tenon’s tissue, a connective tissue surrounding the eyeball, to seal corneal perforations or descemetoceles. It is especially beneficial in emergency settings due to its availability, non-immunogenic nature, and cost-effectiveness. As it is obtained from the patient’s own eye, it eliminates the need for external storage and procurement. TPG can treat corneal perforations that are 2–6 mm in size, and primarily when the defect is secondary to an autoimmune or neuropathic disorder (42). It may be modified or combined with other materials (e.g., amniotic membrane, fibrin glue) to fill in larger defects. Stevens-Johnson Syndrome is specifically listed as one of the autoimmune conditions under “Causative underlying aetiology” for which TPG is indicated. In SJS, ocular surface inflammation, severe dry eye, and corneal melting can ultimately result in corneal perforation—a situation where TPG becomes a viable surgical intervention. Tectonic support can also be achieved using tissue adhesive (cyanoacrylate and fibrin glue) and placing a bandage contact lens thereafter. Cyanoacrylate upon exposure with moisture, polymerizes and exhibits tensile strength. They have a bacteriostatic effect against gram-positive organisms, and they also reduce stromal melting by limiting polymorphonuclear infiltration (43). It can be used as a temporary measure to provide tectonic support for smaller perforations. Its side effects include potential stromal, endothelial and lenticular toxicity upon direct contact with ocular structures, though differentiating these effects from the underlying disease remains challenging.

### 4.2 Tectonic keratoplasty

Conventional large-diameter orthoptic penetrating keratoplasty (PK) can offer anatomic tectonic support in the management of corneal perforations in SJS. However, in patients with severe SJS, such grafts are associated with a higher risk of immune rejection, persistent epithelial defects, secondary infections, graft melt, and eventual recurrence of corneal perforation (44). For this reason, it’s suggestive to use smaller size grafts to seal the perforation and provide support to the corneal tissue. Wang et al. (45) suggested a modified small tectonic keratoplasty technique with minimal corneal graft and conjunctival flap coverage as a globe-preserving option for corneal perforations in severe SJS, reducing the risk of graft rejection, melting and promoting epithelial rehabilitation.

## 5 Visual rehabilitation

### 5.1 Cataract surgery

Patients with SJS have inadequate tear film, misaligned eyelids with trichiasis and scarred, shortened conjunctivae. Cataract formation tends to occur earlier in them due to ongoing inflammation and the prolonged use of steroids. The ocular surface and the persisting inflammation must be addressed before cataract surgery (46).

These eyes have a poor ocular surface; thus, it is suggested to stabilize the ocular surface with preservative-free lubricating agents. The instability of the tear film can also alter the accuracy of preoperative keratometry. Thus, it’s better to perform the surface stabilizing procedure before cataract surgery. In a corneal scar, we can obtain the average keratometry reading of the central 3 mm. In most cases, Barrett universal II can be used, IOL formulas based on artificial intelligence, like Kane and Hill-RBF, are ideal to calculate for flat/steep corneas due to scarring-related irregularities. In significant scarring, standard keratometry values are used (47).

Peribulbar blocks are preferred for most cases, as the compromised media clarity often leads to prolonged surgical time. In eyes with well-preserved corneal clarity, phacoemulsification (Phaco) through a corneal incision is the technique of choice because it causes minimal disruption to the ocular surface. Vasavada and Dholakia emphasized the importance of using controlled parameters in phacoemulsification for total cataracts in patients with SJS. They highlighted that employing the correct technique and staying within the boundaries of the capsulorrhexis was crucial. The authors emphasized the importance of the surgeon’s skill and experience when handling such cases. In the presence of symblepharon and forniceal shortening, a temporal approach was preferable (48). In cases with severe central corneal scarring, we can perform extra capsular cataract extraction (ECCE) via a corneal or limbal incision (2, 49).

Trypan blue is an essential tool to improve visibility during surgery, particularly when a white cataract is present alongside corneal opacity (50). In cases where the visualization is poor, the endoilluminator can be used to enhance the clarity of the capsular margin during phacoemulsification and intraocular lens insertion. The type of intraocular lens we place depends on the type of cataract surgery done. Since the ocular surface, rather than the corneal endothelium, is affected, there is no clear advantage to using a specific irrigating solution or viscoelastic agent during cataract surgery (51). The intraoperative complications observed were typical of any cataract surgery involving compromised media clarity.

Additionally, no postoperative ocular surface breakdown or corneal infection was reported in the study conducted by Iyer et al. (2), while Narang et al. (51) had 4 cases of surface breakdown and 1 case of corneal infection post-cataract surgery. Kasetsuwan et al. (52) identified several factors that may contribute to the worsening of ocular surface disease following cataract surgery. These include the use of topical anesthesia, transection of the corneal nerves, prolonged exposure to intense light from the operating microscope, excessive irrigation of the tear film during surgery, increased inflammatory factors in the tear film due to postoperative ocular surface irritation, and the use of topical medications with preservatives after surgery. Topical non-steroidal anti-inflammatory drugs (NSAIDs) like nepafenac, ketorolac, and diclofenac have been associated with corneal melting, particularly when there is epithelial breakdown; hence, it is better to avoid using NSAIDs in these patients postoperatively (51, 53). If they are on topical or systemic steroids preoperatively, the same should be continued post-surgery as well (51, 54).

With these modifications in mind, useful visual rehabilitation can be achieved.

### 5.2 Keratoprosthesis

Corneal transplantation in patients with severe ocular surface disorder post Steven-Johnson syndrome carries a guarded visual prognosis. Patients undergoing penetrating and lamellar keratoplasty often face complications, including persistent inflammation, non-healing epithelial defects, stromal melting, perforation, and ultimately, graft failure (55). Tugal- Tutkun et al’s study only 2 out of the 7 eyes with SJS achieved a best corrected visual acuity of 20/200 or better (56). Kpro is one of the final resorts for visual rehabilitation in them. Amidst the variable options available, Modified Osteo-odonto keratoprosthesis (MOOKP) is the most employed in such cases. The selection of Kpro depends on the surface status wetness, eyelid function, and the health of the oral mucosa (2, 55).

- Boston keratoprosthesis (type I and II)

Boston keratoprosthesis (Kpro) is the most widely implanted worldwide. Its role in SJS, however is limited. In patients with a severe dry and keratinized ocular surface, Boston type I is unsuitable (55). Boston type II, has been attempted in SJS patients with a better outcome. While visual rehabilitation is possible, long term retention is modest. Complications such as glaucoma progression, retroprosthetic membrane, endophthalmitis, and extrusion is common. Higher cost also limits its use in resource limited settings (57).

- Modified osteo-odonto keratoprosthesis

Modified osteo-odonto keratoprosthesis offers the most consistent long-term outcomes in end stage ocular SJS. Ortiz-Morales et al. reviewed 958 patients (257 SJS), showing 91% visual improvement, 78% achieving >20/400 and a mean anatomic survival of 88% with a follow-up of 30 years (58). SJS patients, although they had higher complication rates, notably mucosal necrosis/ ulceration (43%) and lamina resorption, attributed to persistent mucosal inflammation. Despite this, MOOKP remains the benchmark surgical option in specialized centers with a multidisciplinary team.

- Auro keratoprosthesis and LVP modification

The Auro Kpro, developed in India, is a more cost-effective version of the Boston type I, has been adapted into the LVP Kpro for patients with severe dry eye. The longer optic allows coverage with a mucosal graft, improving suitability for cicatrizing disorders like SJS. The overall retention rates were similar in the Boston Kpro (70.5%) and auroKpro (62.5%). Complications such as intraoperative device breakage (7%) and postoperative extrusions (12.5%) were significantly more common with the auroKpro. The LVP Kpro is for patients with severe dry eye and keratinized ocular surface. It incorporates a longer optical stem that accommodates a protective oral mucosal graft with the corneal carrier graft, thereby creating a stable epithelial surface. In a large series, Basu et al. (59) reported 81% retention at −2.5 years follow-up with significant functional visual recovery in SJS eyes. Compared with Boston type II, the LVP Kpro offers the advantage of a two-stage surgery, better integration with mucosal grafts and markedly lower cost, making it particularly relevant in low-middle-income countries. The same is also true for Auro Kpro; however, the long-term anatomical survival remains lower than that achieved with MOOKP, and complications like mucosal necrosis, device extrusion, and glaucoma progression continue to pose challenges.

- Lucia and Lux Kpro

The Lucia Kpro, designed as a lower cost alternative to the Boston type I, has demonstrated good anatomical survival and functional results in selected cases, though the utility understood in SJS is limited (60). Lux Kpro was specially trialed in India for patients with severe ocular surface disease, including SJS. With its Titanium skirt and extended optic, it has shown promising results. While a longer follow-up is needed, early reports suggest it may serve as a viable alternative to Boston type II in this challenged subgroup (61).

A comprehensive management strategy is crucial as stabilizing the surface may help to prevent corneal blindness. It is imperative to achieve corneal surface stabilization prior to a visual rehabilitative procedure.

## 6 Cost-effectiveness and availability

In resource-limited settings such as India, cost-effectiveness and accessibility are critical determinants of surgical choice for chronic ocular sequelae of SJS. Among stabilization procedures, punctal occlusion and mucous membrane grafting (MMG) are relatively low-cost, require minimal infrastructure, and can be performed widely, making them practical first-line interventions. Amniotic membrane transplantation (AMT) is also moderately cost-effective due to the increasing availability of preserved tissue in eye banks; however, outcomes may be less durable compared to MMG in SJS. By contrast, cultivated oral mucosal epithelial transplantation (COMET), while effective in refractory cases, is associated with high laboratory costs, dependence on specialized culture facilities, and limited availability, restricting its use to select tertiary centers. Minor salivary gland transplantation (MSGT), though surgically more demanding, offers long-term benefits in severe aqueous deficiency and is feasible where surgical expertise exists. Entropion correction using anterior lamellar repositioning with MMG remains relatively inexpensive and widely adaptable. Tectonic procedures, such as Tenon patch grafts (TPG) and tissue adhesives, are inexpensive and particularly valuable in emergencies. At the other end of the spectrum, visual rehabilitation with keratoprosthesis (Boston KPro or MOOKP) and PROSE lenses is highly resource-intensive, requiring specialized centers, long-term follow-up, and significant financial burden, thereby limiting their availability. Overall, MMG, punctal occlusion, and AMT remain the most cost-effective and accessible options in developing countries, whereas advanced techniques like COMET and KPro, although effective, are less feasible due to cost and infrastructure demands.

## 7 Evidence gaps and future directions

A consistent evidence gap across all interventions is the paucity of strong, standardized objective outcome measures, with most studies relying heavily on clinician impressions or nonuniform scales rather than comprehensive biomarker and imaging panels. Rigorous objective tear film analysis is rarely applied comparably across studies. These include tear osmolarity, inflammatory cytokine profiling alongside lipid layer interferometry, noninvasive breakup time, and meibography. Corneal and conjunctival epithelial health is infrequently quantified using standard investigation techniques such as *in vivo* confocal microscopy for nerve density and inflammatory cell counts, impression cytology for goblet cell density and squamous metaplasia grading, and standardized staining scores harmonized to SJS. Graft- or device-specific metrics like goblet cell reconstitution in MMG, epithelial phenotype stability in COMET, limbal niche restoration in SLET, tear secretion rate per unit area and salivary composition vs. basal tear proteome in MSGT, AMT’s anti-fibrotic effect via TGF-*β* pathway readouts, and keratoprosthesis retention with glaucoma structural/functional surveillance—are not uniformly measured. Patient-reported outcomes tailored to SJS (pain, photophobia, dryness, visual function) are sporadically collected and seldom linked to objective parameters. Establishing and adopting an SJS-specific core outcome set that integrates tear biomarkers, epithelial phenotype, nerve metrics, adnexal function, imaging-based inflammation/fibrosis markers, and validated symptom instruments is needed in future.

## 8 Limitations

This review has certain limitations that must be acknowledged. First, the level of evidence available for interventions in chronic ocular Stevens–Johnson syndrome (SJS) is highly heterogeneous, ranging from isolated case reports and small case series to larger observational studies. Randomized controlled trials are scarce, largely due to the rarity of the disease, which poses inherent challenges in conducting adequately powered prospective studies. Second, many of the available studies are retrospective in design and subject to selection bias, variable follow-up durations, and inconsistent outcome measures, limiting the ability to draw definitive conclusions. Third, surgical techniques and postoperative protocols often differ across centers, which introduces heterogeneity and makes direct comparison of results difficult. Finally, most reports originate from tertiary referral centers, which may not fully reflect outcomes in broader clinical practice. These limitations highlight the need for multicenter collaborations and standardized outcome reporting to strengthen the evidence base for managing chronic ocular SJS.

## 9 Conclusion

Chronic ocular Stevens-Johnson Syndrome presents a challenging clinical entity marked by persistent inflammation, progressive scarring, and debilitating sequelae that significantly compromise vision and quality of life. Effective long-term management hinges on a stepwise, tailored strategy aimed at stabilizing the ocular surface, minimizing structural deformities, and facilitating visual rehabilitation.

Tear preservation techniques like punctal occlusion and epithelial restoration procedures such as amniotic membrane transplantation and COMET contribute significantly to maintaining ocular surface health. Minor salivary gland transplantation provides a sustainable solution in patients with severe aqueous deficiency. Surgical correction of cicatricial entropion—common in chronic SJS—must emphasize tissue preservation and anatomical restoration. Anterior lamellar repositioning, with or without mucous membrane grafting, offers functional and symptomatic relief and should be integrated early in the management algorithm.

Visual rehabilitation, particularly in patients undergoing cataract surgery or keratoprosthesis, must be approached cautiously, with adequate preoperative surface optimization to prevent exacerbation of the ocular surface disease. Overall, a comprehensive, multidisciplinary approach tailored to the chronic nature of SJS is essential to achieve long-term ocular surface stability, preserve vision, and improve patient outcomes.

## References

[1] Kanagarajan A Murthy AB Moni PK Palanivel N. Clinicoetiological study of Stevens-Johnson syndrome and toxic epidermal necrolysis Spectrum and the correlation of SCORTEN with prognosis. Indian J Dermatol. (2023) 68:25–33. doi: 10.4103/ijd.ijd_783_22, PMID: 37151260 PMC10162746

[2] Iyer G Srinivasan B Agarwal S Kamala Muralidharan S Arumugam S. Comprehensive approach to ocular consequences of Stevens Johnson syndrome - the aftermath of a systemic condition. Graefes Arch Clin Exp Ophthalmol. (2014) 252:457–67. doi: 10.1007/s00417-014-2568-8, PMID: 24469247

[3] Kaido M Goto E Dogru M Tsubota K. Punctal occlusion in the management of chronic Stevens-Johnson syndrome. Ophthalmology. (2004) 111:895–900. doi: 10.1016/j.ophtha.2003.09.034, PMID: 15121365

[4] Tuberville AW Frederick WR Wood TO. Punctal occlusion in tear deficiency syndromes. Ophthalmology. (1982) 89:1170–2. doi: 10.1016/s0161-6420(82)34659-x, PMID: 7155528

[5] Catt CJ Hamilton GM Fish J Mireskandari K Ali A. Ocular manifestations of Stevens-Johnson syndrome and toxic epidermal necrolysis in children. Am J Ophthalmol. (2016) 166:68–75. doi: 10.1016/j.ajo.2016.03.020, PMID: 27018234

[6] Shanbhag SS Singh S Koshy PG Donthineni PR Basu S. A beginner’s guide to mucous membrane grafting for lid margin keratinization: review of indications, surgical technique and clinical outcomes. Indian J Ophthalmol. (2021) 69:794–805. doi: 10.4103/ijo.IJO_1273_20, PMID: 33727438 PMC8012968

[7] Gurumurthy S Iyer G Srinivasan B Agarwal S Angayarkanni N. Ocular surface cytokine profile in chronic Stevens-Johnson syndrome and its response to mucous membrane grafting for lid margin keratinisation. Br J Ophthalmol. (2018) 102:169–76. doi: 10.1136/bjophthalmol-2017-310373, PMID: 28689166

[8] Pushker N Gorimanipalli B Sharma N Kashyap S Bajaj MS. Mucous membrane grafting (fibrin glue vs. suture) for lid margin pathologies in Stevens-Johnson syndrome: randomized comparative study. Eye (Lond). (2021) 35:1985–92. doi: 10.1038/s41433-020-01203-4, PMID: 33024323 PMC8225623

[9] Iyer G Srinivasan B Agarwal S Pillai VS Ahuja A. Treatment modalities and clinical outcomes in ocular sequelae of Stevens-Johnson syndrome over 25 years--a paradigm shift. Cornea. (2016) 35:46–50. doi: 10.1097/ICO.0000000000000680, PMID: 26555585

[10] Iyer G Pillai VS Srinivasan B Guruswami S Padmanabhan P. Mucous membrane grafting for lid margin keratinization in Stevens–Johnson syndrome: results. Cornea. (2010) 29:146–51. doi: 10.1097/ICO.0b013e3181ae2691, PMID: 20023587

[11] Shanbhag SS Shah S Singh M Bahuguna C Donthineni PR Basu S. Lid-related keratopathy in Stevens-Johnson syndrome: natural course and impact of therapeutic interventions in children and adults. Am J Ophthalmol. (2020) 219:357–65. doi: 10.1016/j.ajo.2020.07.006, PMID: 32681905

[12] Sinha P Nagano H Watanabe A Singh S. Trichiasis in cicatricial ocular surface disease: a multi-center comparison of electroepilation versus eyelash resection outcomes. Orbit. (2024) 43:689–94. doi: 10.1080/01676830.2024.2355639, PMID: 38796788

[13] Tseng SC. Amniotic membrane transplantation for ocular surface reconstruction. Biosci Rep. (2001) 21:481–9. doi: 10.1023/a:1017995810755, PMID: 11900323

[14] Honavar SG Bansal AK Sangwan VS Rao GN. Amniotic membrane transplantation for ocular surface reconstruction in Stevens-Johnson syndrome. Ophthalmology. (2000) 107:975–9. doi: 10.1016/s0161-6420(00)00026-9, PMID: 10811093

[15] Kheirkhah A Ghaffari R Kaghazkanani R Hashemi H Behrouz MJ Raju VK. A combined approach of amniotic membrane and oral mucosa transplantation for fornix reconstruction in severe symblepharon. Cornea. (2013) 32:155–60. doi: 10.1097/ICO.0b013e318247983d, PMID: 22735310

[16] Solomon A Espana EM Tseng SCG. Amniotic membrane transplantation for reconstruction of the conjunctival fornices. Ophthalmology. (2003) 110:93–100. doi: 10.1016/s0161-6420(02)01441-0, PMID: 12511352

[17] Nakamura T Endo K-I Cooper LJ Fullwood NJ Tanifuji N Tsuzuki M . The successful culture and autologous transplantation of rabbit oral mucosal epithelial cells on amniotic membrane. Invest Ophthalmol Vis Sci. (2003) 44:106–16. doi: 10.1167/iovs.02-0195, PMID: 12506062

[18] Kinoshita S Koizumi N Nakamura T. Transplantable cultivated mucosal epithelial sheet for ocular surface reconstruction. Exp Eye Res. (2004) 78:483–91. doi: 10.1016/j.exer.2003.09.004, PMID: 15106927

[19] Nakamura T Yokoo S Bentley AJ Nagata M Fullwood NJ Inatomi T . Development of functional human oral mucosal epithelial stem/progenitor cell sheets using a feeder-free and serum-free culture system for ocular surface reconstruction. Sci Rep. (2016) 6:37173. doi: 10.1038/srep37173, PMID: 27841343 PMC5107917

[20] Nakamura T Takeda K Inatomi T Sotozono C Kinoshita S. Long-term results of autologous cultivated oral mucosal epithelial transplantation in the scar phase of severe ocular surface disorders. Br J Ophthalmol. (2011) 95:942–6. doi: 10.1136/bjo.2010.188714, PMID: 21097786

[21] Ang LPK Nakamura T Inatomi T Sotozono C Koizumi N Yokoi N . Autologous serum-derived cultivated oral epithelial transplants for severe ocular surface disease. Arch Ophthalmol. (2006) 124:1543–51. doi: 10.1001/archopht.124.11.1543, PMID: 17102000

[22] Sotozono C Inatomi T Nakamura T Koizumi N Yokoi N Ueta M . Cultivated oral mucosal epithelial transplantation for persistent epithelial defect in severe ocular surface diseases with acute inflammatory activity. Acta Ophthalmol. (2014) 92:e447–53. doi: 10.1111/aos.12397, PMID: 24835597 PMC4329382

[23] Aziza Y Imai K Itoi M Yoshioka H Komai S Kitazawa K . Strategic combination of cultivated oral mucosal epithelial transplantation and postoperative limbal-rigid contact lens-wear for end-stage ocular surface disease: a retrospective cohort study. Br J Ophthalmol. (2024) 108:1177–83. doi: 10.1136/bjo-2023-323617, PMID: 37918892 PMC11287622

[24] Gopakumar V Agarwal S Srinivasan B Krishnakumar S Krishnan UM Iyer G. Clinical outcome of autologous cultivated Oral mucosal epithelial transplantation in ocular surface reconstruction. Cornea. (2019) 38:1273–9. doi: 10.1097/ICO.0000000000002082, PMID: 31356413

[25] Sotozono C Inatomi T Nakamura T Koizumi N Yokoi N Ueta M . Visual improvement after cultivated oral mucosal epithelial transplantation. Ophthalmology. (2013) 120:193–200. doi: 10.1016/j.ophtha.2012.07.053, PMID: 23084239

[26] Inatomi T Nakamura T Koizumi N Sotozono C Yokoi N Kinoshita S. Midterm results on ocular surface reconstruction using cultivated autologous oral mucosal epithelial transplantation. Am J Ophthalmol. (2006) 141:267–275.e1. doi: 10.1016/j.ajo.2005.09.003, PMID: 16458679

[27] Inatomi T Nakamura T Kojyo M Koizumi N Sotozono C Kinoshita S. Ocular surface reconstruction with combination of cultivated autologous oral mucosal epithelial transplantation and penetrating keratoplasty. Am J Ophthalmol. (2006) 142:757–764.e1. doi: 10.1016/j.ajo.2006.06.004, PMID: 16989763

[28] Nakamura T Inatomi T Sotozono C Amemiya T Kanamura N Kinoshita S. Transplantation of cultivated autologous oral mucosal epithelial cells in patients with severe ocular surface disorders. Br J Ophthalmol. (2004) 88:1280–4. doi: 10.1136/bjo.2003.038497, PMID: 15377551 PMC1772364

[29] Prabhasawat P Chirapapaisan C Ngowyutagon P Ekpo P Tangpagasit W Lekhanont K . Efficacy and outcome of simple limbal epithelial transplantation for limbal stem cell deficiency verified by epithelial phenotypes integrated with clinical evaluation. Ocul Surf. (2021) 22:27–37. doi: 10.1016/j.jtos.2021.06.012, PMID: 34214675

[30] Shanbhag SS Patel CN Goyal R Donthineni PR Singh V Basu S. Simple limbal epithelial transplantation (SLET): review of indications, surgical technique, mechanism, outcomes, limitations, and impact. Indian J Ophthalmol. (2019) 67:1265–77. doi: 10.4103/ijo.IJO_117_19, PMID: 31332106 PMC6677059

[31] Sant’ Anna AEBPP Hazarbassanov RM de Freitas D Gomes JÁP. Minor salivary glands and labial mucous membrane graft in the treatment of severe symblepharon and dry eye in patients with Stevens-Johnson syndrome. Br J Ophthalmol. (2012) 96:234–9. doi: 10.1136/bjo.2010.199901 21527414

[32] Su J-Z Wang Z Liu X-J Lv L Yu G-Y. Use of saliva flow rate measurement in minor salivary glands autotransplantation for treatment of severe dry eye disease. Br J Ophthalmol. (2022) 106:902–7. doi: 10.1136/bjophthalmol-2020-317552, PMID: 33674426 PMC9234421

[33] Vazirani J Bhalekar S Amescua G Singh S Basu S. Minor salivary gland transplantation for severe dry eye disease due to cicatrising conjunctivitis: multicentre long-term outcomes of a modified technique. Br J Ophthalmol. (2021) 105:1485–90. doi: 10.1136/bjophthalmol-2020-316611, PMID: 32938631

[34] Wakamatsu TH SantʼAnna AEBPP Cristovam PC Alves VAF Wakamatsu A Gomes JAP. Minor salivary gland transplantation for severe dry eyes. Cornea. (2017) 36:S26–33. doi: 10.1097/ICO.0000000000001358 28922328

[35] Sharma N Kumar V Bari A Venugopal R Sharma S Agarwal T . The clinical outcomes of minor salivary gland transplantation for severe dry eye disease secondary to chronic Stevens-Johnson syndrome. Ocul Surf. (2024) 34:277–82. doi: 10.1016/j.jtos.2024.08.010, PMID: 39128650

[36] Arboleda A Phansalkar R Amescua G Lee W-S Brandt JD Mannis MJ . Preparing the ocular surface for a Boston Keratoprosthesis type 1 through En bloc minor salivary gland transplantation and mucous membrane grafting in end-stage Stevens-Johnson syndrome. Cornea. (2023) 42:912–6. doi: 10.1097/ICO.0000000000003262, PMID: 37159138 PMC10247429

[37] Anklesaria V Ogbu N Singh S. Long-term outcomes of eyelash-sparing surgical technique for severe segmental cicatricial entropion. Eur J Ophthalmol. (2025) 35:1117–21. doi: 10.1177/11206721241285066, PMID: 39308442

[38] Shree N Das S Arya D Srivastava A Singh A Sangwan V. Single-staged surgical correction of eyelid sequelae along with lid margin mucous membrane grafting in Stevens-Johnson syndrome and other Cicatricial ocular surface diseases. Cornea. (2023) 42:404–11. doi: 10.1097/ICO.0000000000003021, PMID: 35543574

[39] Ross AH Cannon PS Selva D Malhotra R. Management of upper eyelid cicatricial entropion. Clin Experiment Ophthalmol. (2011) 39:526–36. doi: 10.1111/j.1442-9071.2011.02503.x, PMID: 21819506

[40] Singh S Narang P Mittal V. Labial mucosa grafting for lid margin, anterior lamellar, and posterior lamellar correction in recurrent cicatricial entropion. Orbit. (2021) 40:301–5. doi: 10.1080/01676830.2020.1782439, PMID: 32586179

[41] Callahan A. Correction of entropion from Stevens-Johnson syndrome: use of nasal septum and mucosa for severely cicatrized eyelid entropion. Arch Ophthalmol. (1976) 94:1154–5. doi: 10.1001/archopht.1976.03910040066012, PMID: 779733

[42] Korah S Selvin SST Pradhan ZS Jacob P Kuriakose T. Tenons patch graft in the management of large corneal perforations. Cornea. (2016) 35:696–9. doi: 10.1097/ICO.0000000000000808, PMID: 26989954

[43] Deshmukh R Stevenson LJ Vajpayee R. Management of corneal perforations: an update. Indian J Ophthalmol. (2020) 68:7–14. doi: 10.4103/ijo.IJO_1151_19, PMID: 31856457 PMC6951192

[44] Nobe JR Moura BT Robin JB Smith RE. Results of penetrating keratoplasty for the treatment of corneal perforations. Arch Ophthalmol. (1990) 108:939–41. doi: 10.1001/archopht.1990.01070090041035, PMID: 2369351

[45] Wang F Li S Wang T Gao H Shi W. Modified tectonic keratoplasty with minimal corneal graft for corneal perforation in severe Stevens--Johnson syndrome: a case series study. BMC Ophthalmol. (2014) 14:97. doi: 10.1186/1471-2415-14-97, PMID: 25102918 PMC4129433

[46] Sangwan VS Burman S. Cataract surgery in Stevens-Johnson syndrome. J Cataract Refract Surg. (2005) 31:860–2. doi: 10.1016/j.jcrs.2004.07.023 15899470

[47] Priyadarshini K Sharma N Kaur M Titiyal JS. Cataract surgery in ocular surface disease. Indian J Ophthalmol. (2023) 71:1167–75. doi: 10.4103/IJO.IJO_3395_22, PMID: 37026248 PMC10276679

[48] Vasavada AR Dholakia SA. Phacoemulsification in total white cataract with Stevens-Johnson syndrome. Indian J Ophthalmol. (2007) 55:146–8. doi: 10.4103/0301-4738.30713, PMID: 17322609

[49] Sharma N Aron N Venugopal R Sangwan S Titiyal JS Agarwal T. New surgical approach in cases of cataract with ocular Stevens-Johnson syndrome. J Cataract Refract Surg. (2016) 42:1549–55. doi: 10.1016/j.jcrs.2016.09.012, PMID: 27956280

[50] Bhartiya P Sharma N Ray M Sinha R Vajpayee RB. Trypan blue assisted phacoemulsification in corneal opacities. Br J Ophthalmol. (2002) 86:857–9. doi: 10.1136/bjo.86.8.857, PMID: 12140203 PMC1771245

[51] Narang P Mohamed A Mittal V Sangwan VS. Cataract surgery in chronic Stevens-Johnson syndrome: aspects and outcomes. Br J Ophthalmol. (2016) 100:1542–6. doi: 10.1136/bjophthalmol-2015-308041, PMID: 26903523

[52] Kasetsuwan N Satitpitakul V Changul T Jariyakosol S. Incidence and pattern of dry eye after cataract surgery. PLoS One. (2013) 8:e78657. doi: 10.1371/journal.pone.0078657, PMID: 24265705 PMC3827040

[53] Wolf EJ Kleiman LZ Schrier A. Nepafenac-associated corneal melt. J Cataract Refract Surg. (2007) 33:1974–5. doi: 10.1016/j.jcrs.2007.06.043, PMID: 17964407

[54] Sangwan VS Gupta S Das S. Cataract surgery in ocular surface diseases: clinical challenges and outcomes. Curr Opin Ophthalmol. (2018) 29:81–7. doi: 10.1097/ICU.0000000000000441, PMID: 29210839

[55] Sayegh RR Ang LPK Foster CS Dohlman CH. The Boston keratoprosthesis in Stevens-Johnson syndrome. Am J Ophthalmol. (2008) 145:438–44. doi: 10.1016/j.ajo.2007.11.002, PMID: 18207122

[56] Tugal-Tutkun I Akova YA Foster CS. Penetrating keratoplasty in cicatrizing conjunctival diseases. Ophthalmology. (1995) 102:576–85. doi: 10.1016/s0161-6420(95)30980-3, PMID: 7724175

[57] Ament JD Stryjewski TP Pujari S Siddique S Papaliodis GN Chodosh J . Cost effectiveness of the type II Boston keratoprosthesis. Eye (Lond). (2011) 25:342–9. doi: 10.1038/eye.2010.197, PMID: 21183944 PMC3178310

[58] Ortiz-Morales G Loya-Garcia D Colorado-Zavala MF Gomez-Elizondo DE Soifer M Srinivasan B . The evolution of the modified osteo-odonto-keratoprosthesis, its reliability, and long-term visual rehabilitation prognosis: an analytical review. Ocul Surf. (2022) 24:129–44. doi: 10.1016/j.jtos.2022.03.005, PMID: 35314421

[59] Basu S Nagpal R Serna-Ojeda JC Bhalekar S Bagga B Sangwan V. LVP keratoprosthesis: anatomical and functional outcomes in bilateral end-stage corneal blindness. Br J Ophthalmol. (2018) 103:592–8. doi: 10.1136/bjophthalmol-2017-311649 29891734

[60] Ortiz-Morales G Vera-Duarte GR Jimenez-Collado D Rivera JA Rivera KA Domene-Hickman JL . Results of Lucia Keratoprosthesis implantation in severe corneal disease. Am J Ophthalmol. (2024) 268:388–94. doi: 10.1016/j.ajo.2024.08.035, PMID: 39244000

[61] Bakshi SK Graney J Paschalis EI Agarwal S Basu S Iyer G . Design and outcomes of a novel Keratoprosthesis: addressing unmet needs in end-stage Cicatricial corneal blindness. Cornea. (2020) 39:484–90. doi: 10.1097/ICO.0000000000002207, PMID: 31724985

[62] Tseng SCG Di Pascuale MA Liu DT-S Gao YY Baradaran-Rafii A. Intraoperative mitomycin C and amniotic membrane transplantation for fornix reconstruction in severe cicatricial ocular surface diseases. Ophthalmology. (2005) 112:896–903. doi: 10.1016/j.ophtha.2004.11.041 15878073

